# Supplementary material for: SloR-SRE binding to the S. mutans mntH promoter is cooperative
Source: J Bacteriol. 2025 Mar 31;207(5):e00470-24. doi: 10.1128/jb.00470-24 (PMC12096823; doi:10.1128/jb.00470-24)
Supplement: Supplemental figure legends — Legends for Figures S1 and S2. [file jb.00470-24-s0001.docx]

**Supplemental Figure 1.** Binding affinity determinations for the *S. mutans* SloR protein and the *sloABC* and *mntH* promoter probes via biolayer interferometry. Binding measurements were derived from conditions with 5uM SloR protein and a dilution series of DNA target probes ranging from 0.0156uM to 1uM. The real-time binding responses (nM) were recorded over time in seconds (sec) from which the KD value for the interactions was calculated. Shown in (A) is the binding of SloR to a DNA probe spanning 72-bp of the *sloABC* promoter region. Shown in (B) is the binding of SloR to a 90-bp DNA fragment spanning the *mntH* promoter region.

**Supplemental Figure 2.** Binding affinity determinations for the *S. mutans* SloR protein and 22-bp probes in the *mntH* promoter region harboring SRE1 and 2 by biolayer interferometry. Binding measurements were derived from a solution containing 5uM SloR protein and a dilution series of DNA target probes ranging from 0.625uM to 20uM. Real-time binding responses (uM) were recorded over time in seconds (sec) from which KD values were calculated for each interaction. Shown in (A) is the binding interaction between SloR and *mntH* SRE1. (B) reveals no binding curve for SloR-SRE2, indicating no SloR binding to this *mntH* probe.
