# Supplementary figures and images for: SloR-SRE binding to the S. mutans mntH promoter is cooperative
Source: J Bacteriol. 2025 Mar 31;207(5):e00470-24. doi: 10.1128/jb.00470-24 (PMC12096823; doi:10.1128/jb.00470-24)

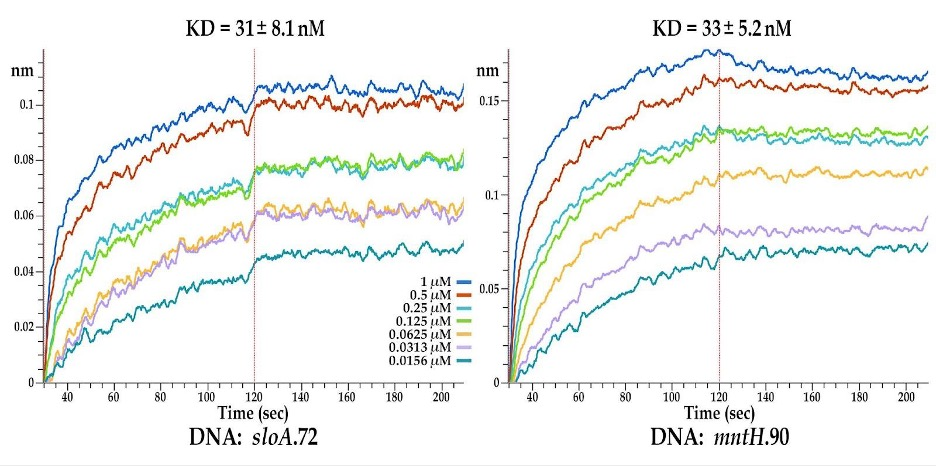

Supplement: Figure S1 — Binding affinity determinations for the S. mutans SloR protein and the sloABC and mntH promoter probes via biolayer interferometry. [file jb.00470-24-s0002.tif]

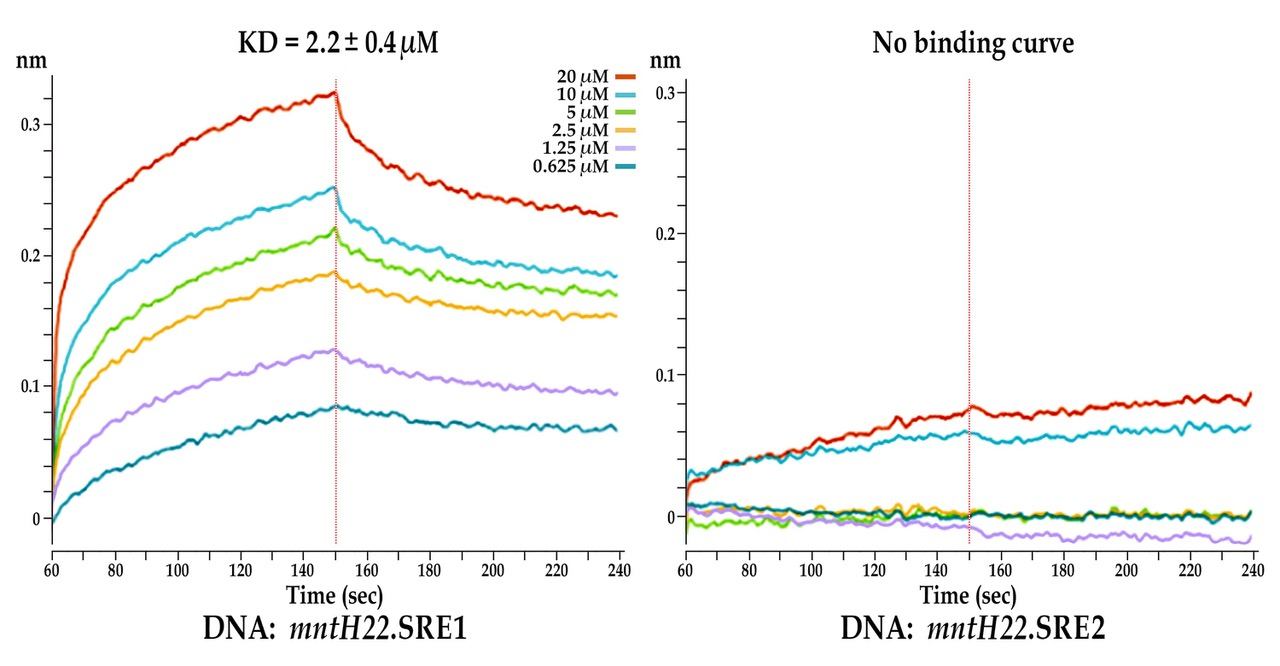

Supplement: Figure S2 — Binding affinity determinations for the S. mutans SloR protein and 22-bp probes in the mntH promoter region harboring SRE1 and 2 by biolayer interferometry. [file jb.00470-24-s0003.tif]
