# Supplementary material for: SloR-SRE binding to the S. mutans mntH promoter is cooperative
Source: J Bacteriol. 2025 Mar 31;207(5):e00470-24. doi: 10.1128/jb.00470-24 (PMC12096823; doi:10.1128/jb.00470-24)
Supplement: TABLE S1 — Primers and oligonucleotides used in this study [file jb.00470-24-s0004.docx]

**Table S1. Primers and oligonucleotides used in this study.**

| **Application** | **Name** | **Nucleotide sequence (5’ to 3’)** | **Annealing temp (°C)** |
| --- | --- | --- | --- |
| **Overlap extension PCR** | **ldhF** | CCGAGCAACAATAACACTC |  |
|  | **ermR** | GAAGCTGTCAGTAGTATACC |  |
|  | **770upF** | ATTACAGTTGCGCCAATGATACC |  |
|  | **770upR-ldh** | GAGTGTTATTGTTGCTCGG*CAATCAGAGCGTTCGTGTTCC |  |
|  | **770dnF-erm** | TATACTACTGACAGCTTC*TAATCAGAGATGTCTAAACAATCAGATTG |  |
|  | **770dnR** | TATTGGCAAACGAAGAGCAAG |  |
| **Mutagenesis primers** | **770.SRE1.mut.P2** | CAGCATATGTTAAGTATGCggAAAAAT |  |
|  | **770.SRE1.mut.P3** | ATTTTTccGCATACTTAACATATGCTG |  |
|  | **700.SRE2.mut.P2** | CATATGTTAAGTATGCTTAAAAATTAAAAAAGGTGTACagATTATTAC |  |
|  | **700.SRE2.mut.P3** | GTAATAATctGTACACCTTTTTTAATTTTTAAGCATACTTAACATATG |  |
|  | **700.SRE1.2.mut.P2** | CATATGTTAAGTATGCggAAAAATTAAAAAAGGTGTACagATTATTAC |  |
|  | **700.SRE1.2.mut.P3** | GTAATAATctGTACACCTTTTTTAATTTTTccGCATACTTAACATATG |  |
| **Real-time**  **semi-qRT-PCR** | **mntH.qRT.F** | GTCTTCACTTATTGCCATGC |  |
|  | **mntH.qRT.R** | GTTGCCATAAGAGCCAATTC |  |
| **Amplicon generation for gel shift** | **mntH.1.R** | CATTTTGAAAATCTCTTTTCTAATATAATTG | 61 |
|  | **mntH.159.F** | TCTCTATTGTGTTTTATACTGTCAGT | 61 |
|  | **mntH.100.F** | AGGTACACCTTTTTTAATTTTTAAGC | 61 |
|  | **mntH.75.R2** | ACCTCGCTGAGTGATTGCTT | 68 |
|  | **mntH.75.F** | GCATACTTAACATATGCTGTTTTTTATGCT | 68 |
|  | **mntH.1.F** | CTTTTCGCAATCTGATTGTTTAG | 61 |
| **Probes in EMSAs**  (top & bottom strand) | **mntH.100.WT** | TACTGTCAGTAATTCTTACAAACTCTTTATATTCAGTAATAATAGGTACACCTTTTTTAATTTTTAAGCATACTTAACATATGCTGTTTTTTATGCTATA  TATAGCATAAAAAACAGCATATGTTAAGTATGCTTAAAAATTAAAAAAGGTGTACCTATTATTACTGAATATAAAGAGTTTGTAAGAATTACTGACAGTA |  |
|  | **mntH.100.CC** | TACTGTCAGTAATTCTTACAAACTCTTTATATTCAGTAATAATAGGTACACCTTTTTTAATTTTTccGCATACTTAACATATGCTGTTTTTTATGCTATA  TATAGCATAAAAAACAGCATATGTTAAGTATGCggAAAAATTAAAAAAGGTGTACCTATTATTACTGAATATAAAGAGTTTGTAAGAATTACTGACAGTA |  |
|  | **mntH.100.CT** | TACTGTCAGTAATTCTTACAAACTCTTTATATTCAGTAATAATctGTACACCTTTTTTAATTTTTAAGCATACTTAACATATGCTGTTTTTTATGCTATA  TATAGCATAAAAAACAGCATATGTTAAGTATGCTTAAAAATTAAAAAAGGTGTACagATTATTACTGAATATAAAGAGTTTGTAAGAATTACTGACAGTA |  |
|  | **mntH.100.CC.CT** | TACTGTCAGTAATTCTTACAAACTCTTTATATTCAGTAATAATctGTACACCTTTTTTAATTTTTccGCATACTTAACATATGCTGTTTTTTATGCTATA  TATAGCATAAAAAACAGCATATGTTAAGTATGCggAAAAATTAAAAAAGGTGTACagATTATTACTGAATATAAAGAGTTTGTAAGAATTACTGACAGTA |  |
|  | **mntH.22.SRE1.WT** | ATTTTTAAGCATACTTAACATA  TATGTTAAGTATGCTTAAAAAT |  |
|  | **mntH.22.SRE1.CC** | ATTTTTccGCATACTTAACATA  TATGTTAAGTATGCggAAAAAT |  |
|  | **mntH.22.SRE1.w10.spcr** | ATTTTTAACCCAATTTAACATA  TATGTTAAATTGGGTTAAAAAT |  |
|  | **mntH.22.SRE1.10IR** | GCAATTAAGCATACTATATTAG  CTAATATAGTATGCTTAATTGC |  |
| **Query sequence from Kajfasz et al. (2020)** | **206-bp sequence upstream of mntH start codon.** | CTTTTCGCAATCTGATTGTTTAGACATCTCTGATTAATTTTCCATAATCTCTATTGTGTTTTATACTGTCAGTAATTCTTACAAACTCTTTATATTCAGTAATAATAGGTACACCTTTTTTAATTTTTAAGCATACTTAACATATGCTGTTTTTTATGCTATAATGCAATTAACCCAATTATATTAGAAAAGAGATTTTCAAAATG | NA |
| **Primers for 5’RACE** | **770.GSP1.2** | TCAACCTACTGTTAGTAGC |  |
|  | **770.GSP2.2** | TGTAAGGCAATCCCTGAGCC |  |
|  | **770_GSP3_nested.2** | TGCCCAGTTTACCAGCCATT |  |
| **Primers for DNase I footprinting** | **mntH.FP.F1** | GATTGTTTAGACATCTCTGATTA |  |
|  | **mntH.FP.R1** | CCTAAAAAAGCTCTTAAAGTTG |  |
| **Probes for Biolayer Interferometry** | **mntH.22.SRE1.WT** | ATTTTTAAGCATACTTAACATA |  |
|  | **mntH.22.SRE2.WT** | AATAATAGGTACACCTTTTTTA |  |
|  | **mntH.90.WT** | GTAATTCTTACAAACTCTTTATATTCAGTAATAATAGGTACACCTTTTTTAATTTTTAAGCATACTTAACATATGCTGTTTTTTATGCTA |  |
|  | **sloA.72.WT** | AGCCTTAATTAATGTAGATTATATTTTTAATTGAACTGAATTAAAAATATAATCCAATATAATGAATATTTT |  |
